# Anemia profile in critical septic patients hospitalized in the ICU

**DOI:** 10.1186/cc9841

**Published:** 2011-03-11

**Authors:** M De la Torre-Prados, A García-de la Torre, J Rodriguez-Vidal, M Nieto-Gonzalez, C Ortiz-García, A García-Alcántara

**Affiliations:** 1 Hospital Virgen de la Victoria, Málaga, Spain; 2 Hospital Reina Sofía, Córdoba, Spain

## Introduction

The aim was to describe the anemia profile of medical or surgical patients with severe sepsis or septic shock in the ICU, assessing severity scale, length of stay and mortality.

## Methods

From January to May 2009, we prospectively selected 79 patients; we excluded hematologic disease. Two groups were established: medical (*n *= 52, 65.8%) and surgical (*n *= 27, 34%) septic patients. The microcytic anemia profile was set in the first 24 hours: CBC, transferrin, serum iron concentration, transferrin saturation index (TSI) and ferritin. There is anemia when haemoglobin (Hb) is <12.5 g/dl (severe when <10 g/dl); mean corpuscular volume (MCV) <80 fl; ferritin <30 ng/ml; TSI <20%; serum iron <50 μg/dl. The reference values of transferrin are 200 to 360 mg/dl. The program used for the data processing and statistical analysis was SPSS.

## Results

The mean age was 60 ± 17 years, 60% were men, APACHE II was 23.46 ± 6.7 and SOFA 9.68 ± 2.93; the length of stay in the ICU was 9.3 ± 5.7 days and 20.3% of mortality. The average values related to microcytic anemia were Hb = 10.02 ± 1.8 g/dl, serum iron = 36 ± 25 μg/dl, MCV = 88.37 fl and TSI = 20.29%. The prevalence of microcytic anemia in our septic patients was 53.8%. In both studied groups we found significant differences in the SOFA (10.17 vs. 8.7, *P *= 0.03) and in transferrin (147 mg/dl vs. 114 mg/dl, *P *= 0.002). The length of stay was higher in the surgical patients (*P *= NS). The mortality showed significant differences in age (58 vs. 66, *P *= 0.03), APACHE II (22 vs. 27, *P *= 0.01), SOFA (9 vs. 12, *P *= 0.0001) and in Hb (10.2 vs. 9.3, *P *= 0.08) and transferrin (141 vs. 117, *P *= 0.02).

## Conclusions

The prevalence of microcytic anemia is more than one-half of our septic patients. There are iron metabolism disorders without significant differences between medical and surgical patients. Transferrin, a protein related to malnutrition, inflammatory response and organ dysfunction, is significantly lower in the most severe patients with higher organ dysfunction scores.

